# Low Body Fat Does Not Influence Recovery after Muscle-Damaging Lower-Limb Plyometrics in Young Male Team Sport Athletes

**DOI:** 10.3390/jfmk5040079

**Published:** 2020-11-05

**Authors:** John F. T. Fernandes, Kevin L. Lamb, Craig Twist

**Affiliations:** 1 Higher Education Sport, Hartpury University, Hartpury GL19 3BE, UK; 2 Department of Sport and Exercise Sciences, University of Chester, Chester CH1 3BJ, UK; k.lamb@chester.ac.uk (K.L.L.); c.twist@chester.ac.uk (C.T.)

**Keywords:** exercise-induced muscle damage, stretch-shortening cycle, fat mass, muscle function

## Abstract

Aim: This study assessed the influence of fat mass to fat-free mass ratio (FM:FFM) on recovery from plyometric exercise. Method: After assessment of body composition, 20 male team sport players (age 20.7 ± 1.1 years; body mass 77.1 ± 11.5 kg) were divided into low- (*n* = 10; 0.11 ± 0.03) and normal- (*n* = 10; 0.27 ± 0.09) fat groups based on FM:FFM ratio. Thereafter, participants completed measurements of knee extensor torque at 60 and 240°∙s^−1^, countermovement jump flight time, plasma creatine kinase (CK) activity and perceived muscle soreness (VAS) before and at 0, 24 and 48 h after 10 × 10 maximal plyometric vertical jumps. Results: Evidence of muscle damage was confirmed by alterations in VAS, peak torque at 60 and 240°∙s^−1^ and flight time at 0, 24 and 48 h after plyometric exercise (*P* < 0.05). CK was increased at 0 and 24 h (*P* < 0.05) but returned to baseline values by 48 h. No time by group effects were observed for any of the dependent variables (*P* > 0.05). Conclusion: The current findings indicate that while muscle damage was present after plyometric exercise, the magnitude was similar across the two body composition groups. Applied practitioners can allow for a similar recovery time after plyometric exercise in those with low and normal body fat.

## 1. Introduction

The stretch-shortening cycle underpins the mechanistic basis of plyometric exercise [1] that is widely used to advance athletic performance [2] and forms a natural part of many sporting movements. Indeed, plyometric training can provide a range of performance-related benefits, including improvements in vertical jump height [3], sprint performance [4], improved running economy [5] and skill-related actions [6].

A consequence of such training, however, particularly when it is unaccustomed or comprising high volume, is damage to the muscle ultra-structure [7]. The mechanisms of exercise-induced muscle damage (EIMD), which have been discussed extensively elsewhere [8,9], include mechanical (e.g., ‘popping’ sarcomere hypothesis, impairments in excitation-contraction coupling) and metabolic (e.g., alterations in metabolic rate, glycogen depletion) disturbances. Notwithstanding its mechanisms, the symptoms of EIMD include increases in muscle soreness, intramuscular enzymes in the blood, and, of most importance to the athlete, an impaired muscle function that includes reductions in muscle strength and power [9,10]. These symptoms typically peak between 24 and 48 h after the initial bout and are recovered by seven days [9,10].

Although the acute indicators of EIMD are well-documented, little is known about the factors that exacerbate the associated symptoms and the subsequent recovery from this bout. Importantly, the appearance of these symptoms is not synchronous and often highly individualized [11,12]. One factor that might exacerbate symptoms of EIMD is the vertical loading on the muscle caused by the individual’s body composition. Whilst lean tissue is functional and contributes to force-generating capacity, adipose tissue does not. Vertical loading is also known to be greater in obese compared to non-obese individuals [13].

It stands that body fat might act as a ‘vacant’ load on the working muscles and has been shown to intensify symptoms after muscle-damaging exercise [14,15]. Hickner and colleagues reported that those with a higher fat-to-fat-free mass ratio (FM:FFM) experienced greater losses in muscular strength after downhill running [14]. Similarly, in the absence of any vertical loading (i.e., during knee extension exercise), Paschalis and colleagues [15] observed a greater magnitude of EIMD in overweight versus underweight females that might be influenced by cytokine release and insulin resistance from greater adipose tissue [14].

Weak associations have also been reported between FM:FFM ratio and quadricep force loss after prolonged intermittent running in rugby players [16]. However, dissimilar patterns of vertical loading between downhill and intermittent shuttle running coupled with a homogenous group with similar body fat mass might explain these weak associations. Further work using damaging exercise with a greater vertical component (e.g., plyometric exercise) in a more heterogeneous group is therefore warranted. A study of this kind would have clear implications for those using damaging exercise with groups of individuals whose body composition varies. The purpose of this study was to assess the influence of fat mass, using FM:FFM ratio, and the rate of recovery of indirect markers of exercise-induced muscle damage after plyometric exercise.

## 2. Materials and Methods

### 2.1. Participants

Twenty recreational male team sport players (10 per group; age 20.7 ± 1.1 years; body mass 77.1 ± 11.5 kg; FM:FFM ratio 0.19 ± 0.10) were recruited for the study. All participants were asymptomatic of illness and injury, and to negate the effects of a prior bout of EIMD, participants had not performed systematic lower-limb training in the six months beforehand [17]. Participants provided written consent for the study, which was approved by the Ethics Committee of the host institution (SES121301; 26 June 2012). Participants were instructed not to consume any ergogenic supplements (for example, caffeine) on the day of testing and to refrain from exercise, other than that performed as part of the study, throughout their involvement.

### 2.2. Study Design

**Figure 1:**
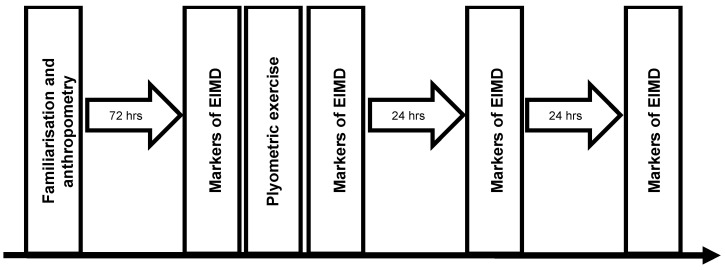
Schematic of study design.

The study used a repeated measures design in which participants attended the laboratory on four occasions (Figure 1). On the first visit participants had their body composition assessed and were habituated to measures of muscle soreness, isokinetic knee extensor strength and maximal countermovement jump. Participants returned to the laboratory three days later for baseline measurements of plasma creatine kinase (CK) activity, muscle soreness, isokinetic extensor strength and maximal countermovement jump. Repeated measurements were conducted at 0, 24 ± 1 and 48 ± 1 h after muscle-damaging plyometric exercise (10 × 10 countermovement jumps).

### 2.3. Anthropometric Measurements

The BodPod (S/T, Life Measurement Inc., Concord, CA, USA) was used as it is valid and non-invasive method to measure body composition [18], that demonstrates a low technical error and high internal validity [19]. This device employs air displacement plethysmography to measure body volume and subsequently body density. Thereafter, body fat percentage was estimated using the Siri equation from which body fat mass (FM; kg), fat-free mass (FFM; kg) and fat mass: fat free mass (FM:FFM; fat mass divided by fat-free mass) were calculated. Participants were divided into a “low fat” (*n* = 10) and “normal fat” group (*n* = 10) for analysis based upon a median split of FM:FFM ratio. The terms and values “low fat” and “normal fat” are consistent with previous worked [14] and reference population data [20].

### 2.4. Assessment of Perceived Muscle Soreness

Perceived muscle soreness of the knee extensors was measured using a visual analogue scale (VAS). The VAS is numbered from 0 to 10 (on the concealed reverse side of the scale) where 0 indicates “no soreness on movement” and 10 indicates “muscles are too sore to move”. With feet shoulder width apart and hands on hips, participants descended until their hips were below their knee joint at which point the VAS was held up for them to indicate their rating of perceived soreness on the continuum. This method has been used previously as an indirect marker of muscle damage [21].

### 2.5. Assessment of Plasma Creatine Kinase Activity

Plasma CK activity was determined from a capillary blood sample of the participant’s preferred finger. A 30 µl sample of whole blood was collected into a heparinised capillary tube and pipetted onto a test strip for analysis (Reflotron, Type 4, Boehringer Mannheim, Mannheim, Germany). The Reflotron employs a photometric process to determine CK activity. This procedure has been used previously to confirm tissue damage after exercise [21].

### 2.6. Assessment of Knee Extensor Isokinetic Strength

A dynamometer (Biodex, Multi-joint system 3, Biodex Medicial, New York, NY, USA) was used to measure isokinetic strength of the dominant knee extensors at velocities of 60°∙s^−1^ and 240°∙s^−1^. To prevent extraneous body movements, Velcro straps were applied tightly across the chest and thigh. Participants were provided with strong verbal encouragement and real-time feedback via the computer’s monitor. Limb mass was calculated to enable gravitational correction and range of motion was set for each participant. Participants performed five repetitions at each speed, with the slower velocity employed first, to improve reliability, [22] and two minutes rest between sets [23]. The peak (i.e., highest value across the five repetitions) torque values were recorded and used for analysis.

### 2.7. Assessment of Maximal Vertical Jump Height

Maximal countermovement jump flight time was recorded using an infra-red timing system (Optojump, Microgate S.r.l., Bolzano, Italy). With their hands-on hips, participants squatted down into a self-selected depth before explosively performing the concentric action. Participants performed three maximal countermovement jumps with one-minute rest between each jump. This method has been used previously as an indirect marker of EIMD [24] and is a reliable marker of jump performance [25].

### 2.8. Muscle-Damaging Protocol

The muscle-damaging exercises comprised 10 sets of 10 maximal countermovement jumps with 60 s rest between sets. Upon landing, participants were instructed to assume a 90° angle at the knee joint before the next jump. During the protocol, an infra-red timing system was used to record power output (Optojump, Microgate S.r.l., Bolzano, Italy). At the end of each set, participants were provided with feedback regarding their jump performance to encourage maximal effort. The same method has successfully induced muscle damage in previous studies [23,26].

### 2.9. Statistical Analysis

All data collected were analyzed using SPSS (version 24, IBM SPSS Inc, Chicago, IL, USA). Assumptions of normal distribution and homogeneity of variance were checked via Shapiro Wilks and Levene statistics, respectively, and repeatedly found to be satisfied. An independent samples *t*-test was employed to compare anthropometric characteristics between groups. Thereafter, a two-way (time × fat group) repeated measures analysis of variance tested the null hypothesis of no difference between the two groups. Following a violation of Mauchly’s test of Sphericity (*P* < 0.05), the Greenhouse–Geisser correction was used. A post hoc t-test was conducted to locate the differences between levels of the dependent variables, with a Tukey LSD correction [27]. Finally, effects sizes (ES) (Cohen’s d; difference between the means divided by the pooled standard deviation) and 90% confidence intervals were calculated to determine the size of the differences. The following quantitative criteria were used to explain the practical significance of the findings: trivial < 0.2, small 0.2–0.6, moderate > 0.6–1.2, large > 1.2–2.0, and very large > 2.0. Alpha was set at 0.05.

## 3. Results

### 3.1. Anthropometric Characteristics

There were no differences between groups for body mass (*t* = −0.1, *P* = 0.951, ES = 0.05 ± 1.73). However, fat-free mass was greater in the low-fat group (*t* = 2.6, *P* = 0.020, ES = −1.13 ± 0.94), whereas the normal-fat group had higher fat mass (*t* = −3.3, *P* = 0.004, ES = 4.50 ± 3.14), body fat percentage (*t* = −5.6, *P* < 0.001, ES = 3.69 ± 1.41) and FM:FFM ratio (*t* = −4.9, *P* < 0.001, ES = 4.24 ± 1.91) than the low-fat group. All anthropometric data are shown in Table 1.

### 3.2. Muscle-Damaging Protocol

There were no differences in average flight time during the plyometric jumps between the low-fat (0.49 ± 0.03 s) and normal-fat (0.46 ± 0.06 s) groups (*t* = 1.1, *P* = 0.257, ES = 0.73 ± 1.32).

### 3.3. Plasma Creatine Kinase Activity

**Figure 2:**
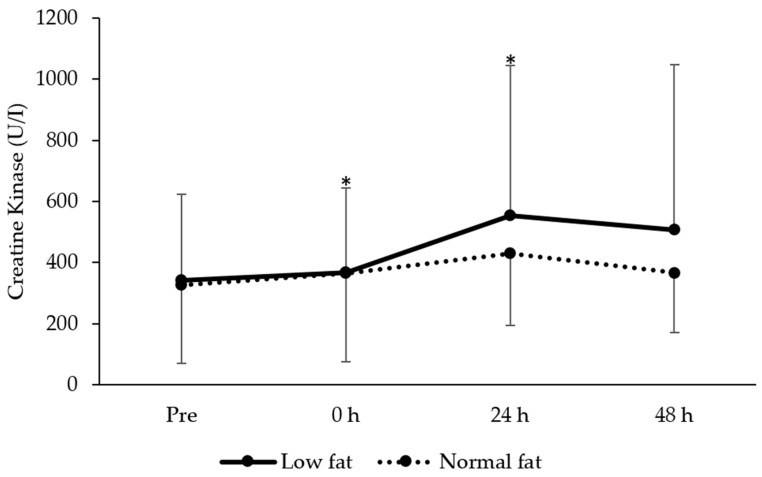
Changes in creatine kinase (mean ± standard deviation) between low- and normal-fat groups at pre, 0, 24 and 48 h after resistance exercise. * denotes significantly different from pre for the whole sample (*P* < 0.05).

A main effect of time was revealed for CK activity after plyometric exercise (F = 4.0, *P* = 0.036), for which post hoc analysis revealed increases in plasma CK at 0 (*t* = −2.8, *P* = 0.011 ES = 0.12 ± 0.63) and 24 h (*t* = −3.2, *P* = 0.005, ES = 0.58 ± 0.77) after plyometric exercise (*P* < 0.05). There was no group (F = 0.5, *P* = 0.509) or time × group interaction (F = 0.7, *P* = 0.743) for CK activity. Changes in CK activity are shown in Figure 2.

### 3.4. Perceived Muscle Soreness

**Figure 3:**
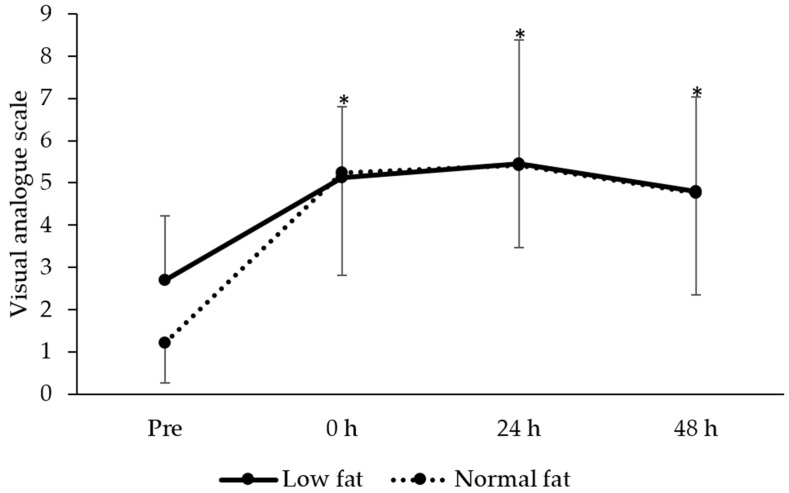
Changes in perceived muscle soreness (mean ± standard deviation) between low- and normal-fat groups at pre, 0, 24 and 48 h after resistance exercise. * denotes significantly different from pre for the whole sample (*P* < 0.05).

There was a main effect of time on perceived muscle soreness (F = 15.6, *P* < 0.001; Figure 3), with post hoc analysis revealing muscle soreness was higher at 0 h (*t* = −6.9, *P* < 0.001, ES = 2.13 ± 0.75), 24 h (*t* = −6.3, *P* < 0.001, ES = 2.31 ± 0.85) and 48 h (*t* = −4.4, *P* < 0.001, ES = 1.87 ± 0.83) after the muscle-damaging protocol (*P <* 0.05). There was no group (F = 0.1, *P* = 0.752) or time × group interaction for perceived muscle soreness (F = 1.1, *P* = 0.354).

### 3.5. Knee Extensor Isokinetic Strength

**Figure 4:**
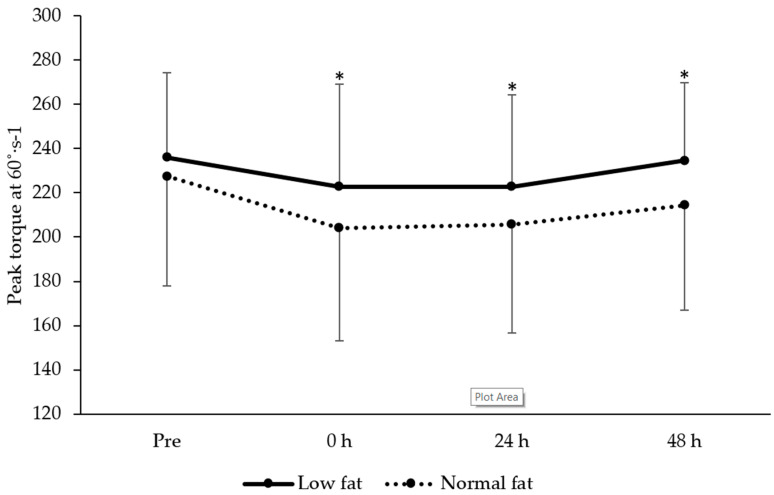
Changes in knee extensor peak torque at 60°∙s^−1^ (mean ± standard deviation) between low- and normal-fat groups at pre, 0, 24 and 48 h after resistance exercise. * denotes significantly different from pre for the whole sample (*P* < 0.05).

**Figure 5:**
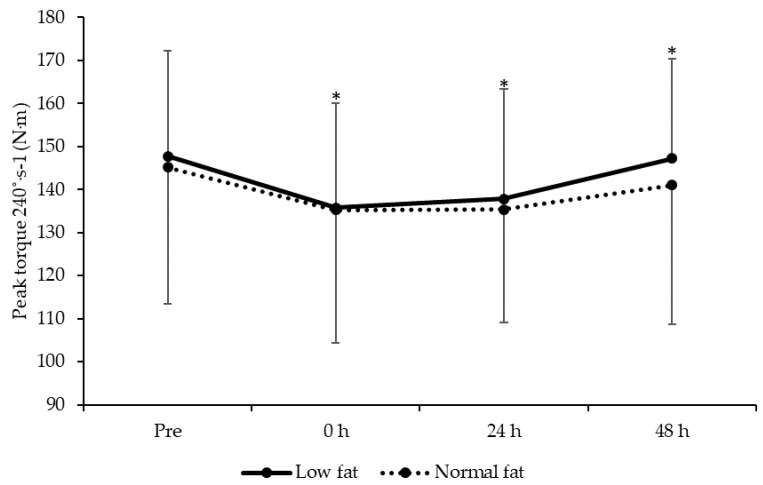
Changes in knee extensor peak torque at 240°∙s^−1^ (mean ± standard deviation) between low- and normal-fat groups at pre, 0, 24 and 48 h after resistance exercise. * denotes significantly different from Pre for the whole sample (*P* < 0.05).

A main effect of time on peak torque at 60 and 240°∙s^−1^ was observed (F = 7.6, *P* < 0.001 and F = 7.3, respectively, *P* < 0.001). After plyometric exercise peak torque for 60°∙s^−1^ was lower than baseline at 0 (*t* = 4.9, *P* < 0.001, ES = −0.41 ± 0.65), 24 (*t* = 5.5, *P* < 0.001, ES = −0.39 ± 0.63) and 48 h (*t* = 3.1, *P* = 0.006, ES = −0.18 ± 0.63). Similarly, peak torque at 240°∙s^−1^ was lower at 0 (*t* = 4.4, *P* < 0.001, ES = −0.38 ± 0.71), 24 (*t* = 3.6, *P* = 0.002, ES = −0.34 ± 0.59) and 48 h (*t* = 2.3, *P* = 0.033, ES = −0.09 ± 0.62) after plyometric exercise. However, there was no group (60°∙s^−1^: F = 1.4, *P* = 0.255; 240°∙s^−1^: F = 0.6, *P* = 0.469) or time × group interaction (60°∙s^−1^: F = 0.9, *P* = 0.451; 240°∙s^−1^: F = 0.5, *P* = 0.693) effects. Data for knee extension after plyometric exercise are shown in Figure 4 and Figure 5.

### 3.6. Maximal Vertical Jump Performance

**Figure 6:**
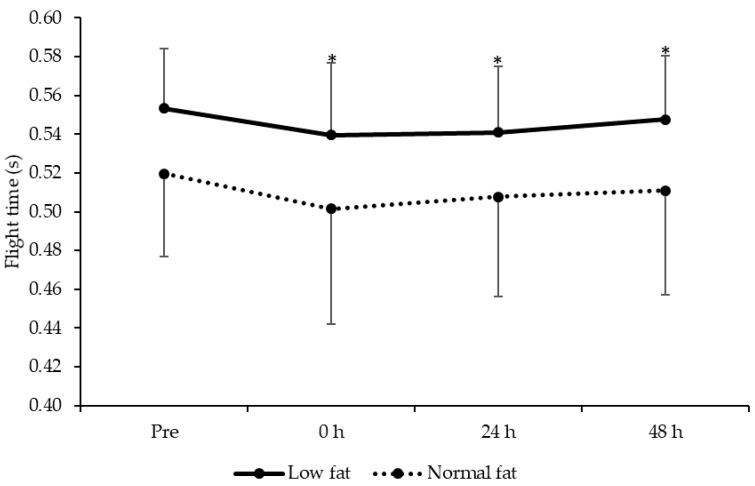
Changes in flight time (mean ± standard deviation) between low- and normal-fat groups at pre, 0, 24 and 48 h after resistance exercise. * denotes significantly different from pre for the whole sample (*P* < 0.05).

A main effect of time on flight time was observed (F = 3.7, *P* = 0.019, Figure 6) with post hoc analysis revealing that flight time was impaired at 0 (*t* = 2.7, *P* = 0.014, ES = −0.38 ± 0.71), 24 (*t* = 2.5, *P* = 0.022, ES = −0.29 ± 0.66) and 48 h (*t* = 2.6, *P* = 0.020, ES = −0.22 ± 0.70) after plyometric exercise. There was no group (F = 4.0, *P* = 0.064) or time × group (F = 0.2, *P* = 0.900) interaction effects.

## 4. Discussion

The study sought to determine the influence of fat mass, using FM:FFM ratio, on recovery after muscle-damaging plyometric exercise. For the first time, we report that despite immediate and prolonged symptoms of exercise-induced muscle damage, reductions in force-generating capacity, muscle soreness and CK activity are similar irrespective of an individual’s FM:FFM ratio. These findings are therefore in contrast to previous studies that have reported the muscle damage response to be dependent on an individual’s body fat content [14,15].

Alterations in muscle soreness, circulating CK and muscle functional markers (peak torque and flight time) provide indirect evidence of muscle damage after plyometric exercise [10,28]. Notably, the reductions in peak torque (~9.4 to 12.5%) and flight time (~5.1%) after plyometric exercise (at 0 to 48 h) were similar to other studies using plyometric exercise (e.g., ~9.6 to 13%) [29,30], but modest in comparison to other muscle-damaging modalities, e.g., ~27% for downhill running [31]. We reaffirm that muscle damage incurred after plyometrics is relatively ‘mild’ when compared to more severe forms of damaging exercise (e.g., resistance exercise, downhill running) [32], which is confirmed by the relatively small effects sizes reported.

In studies that showed those with higher body fat experienced a greater muscle damage [14,15], muscle-damaging exercise was more severe (downhill running and resistance exercise, respectively). The modest responses to plyometric exercise observed herein is the most likely candidate for why body fat did not influence the magnitude of muscle damage incurred in participants. Moreover, previous work has suggested that cytokines released from adipose tissue might contribute to greater symptoms of EIMD in those with a higher FM:FFM ratio [14]. Despite the differences in fat mass between groups, our findings dispute those of Hickner and colleagues’ suggestions. Again, the modest EIMD to plyometric exercise is pertinent in that the severity might not be sufficient to induce a cytokine response [8]. We contest that the FM:FFM ratio might not influence recovery when the magnitude of resultant muscle damage from exercise is small.

We originally postulated that differences in vertical loadings between exercise modalities (e.g., downhill running vs shuttle running) and the homogeneity of the groups (in terms of their fat mass) might have explained why studies had [14] and had not [16] observed an effect of body composition on muscle damage response. However, the loading during maximal countermovement jump exercise was largely vertical and the range in fat mass of our participants (32.7 kg) was substantially greater than previous work (7.4–15 kg). Consequently, we propose that neither the vertical loadings nor range of fat mass values explains the discrepancy in the current literature on body composition and recovery.

There is a suggestion that the external load during exercise is related to the fatigue and recovery response. For example, Marginson and colleagues [33] reported that a higher power output during plyometric jumps resulted in greater decrements in muscular strength and countermovement jump height. Similarly, those expressing a higher power output during squatting exercise were subject to greater losses in knee extensor force and lower-body power immediately after exercise [34]. The findings of the current study would support this notion in that both groups maintained similar jump performances (i.e., average flight time) during plyometric exercise and were consequently subject to the same fatigue (at 0 h) and muscle damage response (at 24 and 48 h). Importantly, this study suggests that those with a higher FM:FFM ratio are subject to a similar post-plyometric exercise alteration as those with a lower FM:FFM ratio, providing they maintain a similar external load.

One limitation of the current study is the failure to match the groups for lean mass. This might suggest that the findings cannot wholly be attributed to differences in fat mass. However, the groups were matched for body mass, with the composition of that mass being different between groups, i.e., a higher fat-free mass and lower fat mass in the low-fat than the normal-fat group. Thus, the comparable magnitude of muscle damage observed in this study can still be attributed to differences in body composition.

## 5. Conclusions

The current findings indicate that while modest symptoms of exercise-induced muscle damage were present after plyometric exercise, an individual’s FM:FFM ratio did not influence the magnitude of this response. Thus, applied practitioners can structure vertically loaded plyometric training of team sport athletes with low and normal body fat in a similar manner. Furthermore, that four of the five muscle damage markers did not return to baseline values by 48 h means that training in the days after novel plyometric exercise should be structured accordingly to allow for the extended recovery time. That is, more than 48 h recovery after lower-body plyometric exercise is needed should applied practitioners wish for their athletes to exercise without muscle soreness or compromised muscle function.

## Figures and Tables

**Table 1:** Anthropometric characteristics (mean ± standard deviation) and comparison of low- and normal-fat groups.

Characteristic	Low Fat (*n* = 10)	Normal Fat (*n* = 10)
Body mass (kg)	76.9 ± 6.1	77.2 ± 15.5
Fat mass (kg)	8.0 ± 1.8	16.7 ± 8.2 *
Fat-free mass (kg)	68.6 ± 6.6	60.4 ± 7.7 *
Body fat (%)	10.6 ± 2.5	20.8 ± 5.1 *
FM:FFM ratio	0.12 ± 0.03	0.27 ± 0.09 *

* denotes significantly different between groups (*P* < 0.05).
